# Taking Care of Us© (TCU) study protocol: feasibility and acceptability of a dyadic intervention for couples living with heart failure

**DOI:** 10.1186/s40814-023-01249-7

**Published:** 2023-01-25

**Authors:** Karen S. Lyons, Carol J. Whitlatch, Amanda R. Vest, Jenica N. Upshaw, Stacy Hutton Johnson, Jeremiah Morelock, Christopher S. Lee

**Affiliations:** 1 Boston College William F. Connell School of Nursing, Maloney Hall, 231, 140 Commonwealth Avenue, Chestnut Hill, MA 02467 USA; 2 Center for Research and Education, Benjamin Rose Institute On Aging, Cleveland, OH USA; 3 Cardiac Transplantation Program, Tufts Medical Center, Boston, MA USA; 4 Tufts University School of Medicine, Boston, MA USA; 5 Cardio-Oncology Program| Division of Cardiology, Tufts Medical Center, Boston, MA USA; 6 Canopy Consultants Inc., Arlington, MA USA; 7 Australian Catholic University Mary MacKillop Institute for Health Research, Melbourne, Australia

**Keywords:** Heart failure, Couples, Dyadic health, Communication, Collaboration, Support, Physical health, Mental health, Zoom-based

## Abstract

**Background:**

There are more than 1 million hospital admissions and 3 million emergency visits for heart failure in the USA annually. Although spouse/partners make substantial contributions to the management of heart failure and experience poor health and high levels of care strain, they are rarely the focus of heart failure interventions. This protocol describes a pilot randomized controlled trial that tests the feasibility, acceptability, and preliminary change in outcomes of a seven-session couple-based intervention called *Taking Care of Us©* (TCU). The TCU© intervention is grounded in the theory of dyadic illness management and was developed to promote collaborative illness management and better physical and mental health of adults with heart failure and their partners.

**Methods:**

A two-arm randomized controlled trial will be conducted. Eligible adults with heart failure and their co-residing spouse/partner will be recruited from a clinical site in the USA and community/social media outreach and randomized to either the TCU© intervention or to a control condition (SUPPORT©) that offers education around heart failure management. The target sample is 60 couples (30 per arm). TCU© couples will receive seven sessions over 2 months via Zoom; SUPPORT© couples will receive three sessions over 2 months via Zoom. All participants will complete self-report measures at baseline (T1), post-treatment (T2), and 3 months post-treatment (T3). Acceptability and feasibility of the intervention will be examined using both closed-ended and open-ended questions as well as enrollment, retention, completion, and satisfaction metrics. Preliminary exploration of change in outcomes of TCU© on dyadic health, dyadic appraisal, and collaborative management will also be conducted.

**Discussion:**

Theoretically driven, evidence-based dyadic interventions are needed to optimize the health of both members of the couple living with heart failure. Results from this study will provide important information about recruitment and retention and benefits and drawbacks of the TCU© program to directly inform any needed refinements of the program and decision to move to a main trial.

**Trial registration:**

ClinicalTrials.gov (NCT04737759) registered on 27 January 2021.

## Background

Heart failure is the fastest growing cardiovascular disorder in the USA and the most common reason for both hospitalization and rehospitalization among older adults [1, 2]. Adults with heart failure experience severe symptom burden, significant functional limitations, and poor quality of life [3, 4]. Although family care partners (e.g., spouse or adult–child) make substantial contributions to the management of heart failure [5], experience poor health themselves [6] and significant care strain [7], they are rarely the focus of heart failure interventions [8–10]. Thus, there is great need for novel theoretically informed interventions targeted at the heart failure dyad (i.e., adult with heart failure and their care partner) to reduce the family burden of heart failure [11–13].

Heart failure is a clinical diagnosis based on a history of symptoms and physical examination [14]. Adults with heart failure experience considerable variability in symptoms and changes in symptoms are the primary reason why adults with heart failure seek treatment [15, 16]. Thus, symptoms are critical elements of the diagnosis, management, and the lived-experience of heart failure for both the person with the illness and also their care partner. The continual need for symptoms to be managed place considerable demands on heart failure care dyads, resulting in poor physical and mental health for both members [17–22].

Most non-pharmacologic heart failure interventions involve patient education and behavioral counseling, with limited success [10, 23]. Potential reasons for lack of efficacy include a focus on adherence behaviors, interventions that do not include family members or care partners, and failure to acknowledge the interpersonal context of illness [24, 25]. Similarly, 50% of the small number of heart failure interventions targeting the care partner did not improve outcomes [8]. Within the broader chronic illness literature, dyad-based interventions have been found to be more successful than individual interventions [26, 27]. Yet, despite recent movement to involve care partners in heart failure interventions [28], the focus has been predominantly on adherence behaviors and education, demonstrating inconsistent efficacy on outcomes for the dyad [10]. A review of interventions in coronary artery disease dyads highlighted the effectiveness of targeting psychosocial variables [29], with communication and positive interpersonal dynamics considered essential for improving the outcomes of both the adult with heart failure and their care partner [24, 25, 28].

Across illness contexts, there is strong evidence of the interdependent nature of the experience within couples and the important roles of support and a sense of “we-ness” [30–36]. Building on this salient work and the fields of illness management and caregiving, the guiding theory for the *Taking Care of Us©* (TCU) program, the theory of dyadic illness management [31], focuses on the couple as the unit of interest with the main goal to optimize health within the couple by balancing the needs of both members. The theory proposes that couples who have shared appraisal about the illness experience (e.g., similar appraisals of the person with heart failure’s symptoms) are more likely to engage in dyadic illness management behaviors (e.g., collaborative symptom management behaviors, open communication about the illness, shared health activities). Dyadic management encompasses the spectrum of how couples *collaborate*, *communicate* and feel *confident* around management of the illness. Greater shared appraisal and engagement in dyadic management behaviors lead to better physical and mental health of both members of the couple. Thus, TCU© focuses on the concepts of dyadic symptom appraisal, dyadic management behaviors and dyadic health.

Shared dyadic appraisal is vital to the effective management of heart failure within couples [37]. Studies have found moderate gaps in appraisal of heart failure symptoms within the couple [38–40], which has been associated with inadequate illness management, lower levels of collaboration, and poor outcomes [41–44]. Yet, no intervention has targeted symptom appraisal within the context of the couple. Similarly, research has found collaborative verbal and non-verbal behaviors to manage symptoms and illness are associated with better health outcomes [45–49], with emerging evidence in heart failure [19, 50–52]. Finally, confidence (self-efficacy) plays an important role in health behavior change [53] and is a defining characteristic of self-management programs [54, 55]. Within heart failure, confidence has been associated with greater engagement in management behaviors and better health outcomes for the adult with heart failure and care partner [56–63]. Yet, few heart failure studies have examined confidence of both members of the couple or capitalized on the known social context of confidence [62].

**Fig. 1:**
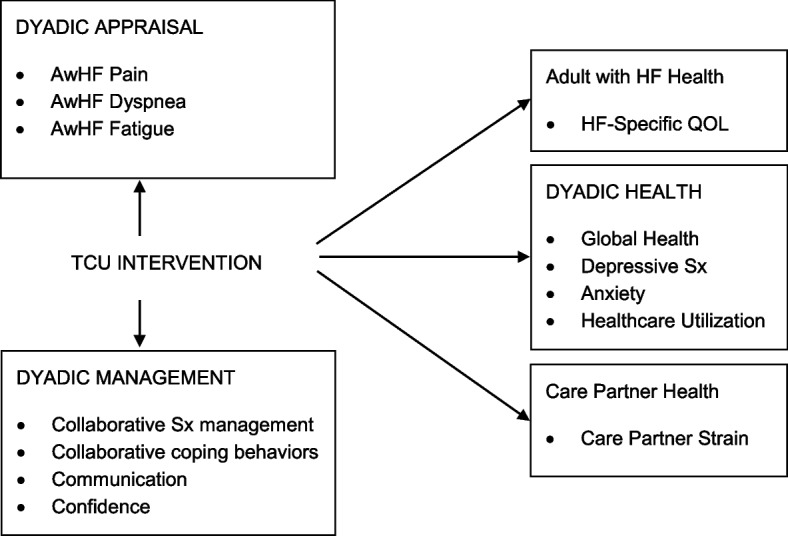
Conceptual framework

To address these gaps, we designed a theoretically informed and evidence-based dyadic intervention for adults with heart failure and their spouse/partner, *Taking Care of Us©* (Fig. 1). The aims of this pilot study were to (1) determine feasibility and acceptability of the intervention to inform the decision to move to a main trial and (2) explore preliminary change in outcomes of the intervention, namely dyadic health (i.e., global health, depressive symptoms, anxiety, and healthcare utilization), dyadic symptom appraisal (i.e., pain, dyspnea, fatigue), and dyadic management behaviors (i.e., collaborative symptom management, collaborative coping behaviors, communication, and confidence).

## Methods

### Design

**Fig. 2:**
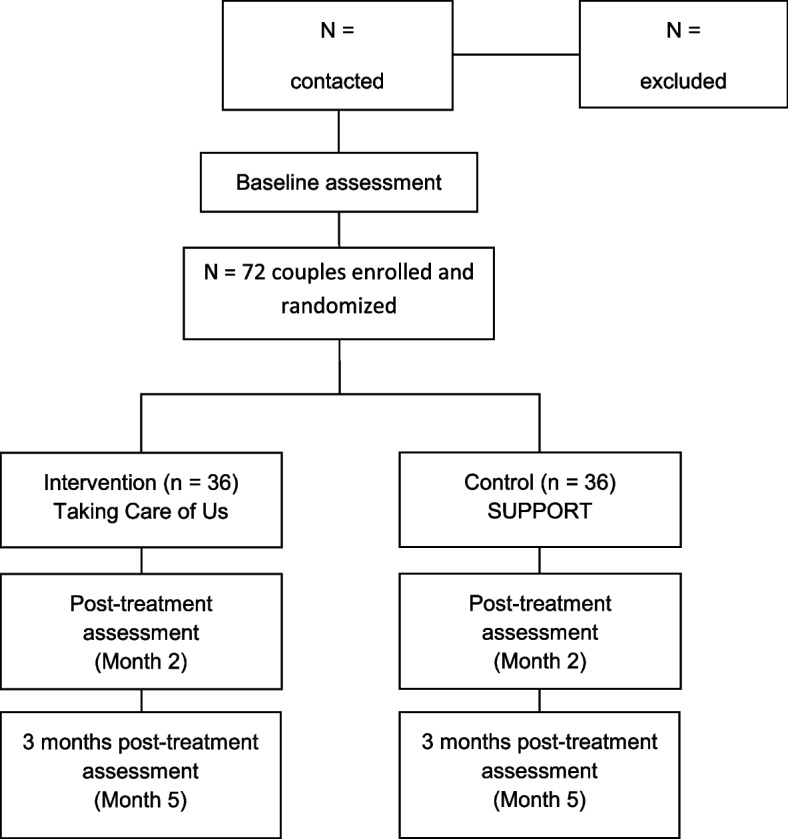
Study flow diagram

This is a two-arm, pilot randomized controlled trial comparing the TCU© program with an educational counseling attention-control condition, SUPPORT©. A target of 72 couples will be enrolled in the study, which allows for 17% attrition to achieve a complete sample of 60 couples. Couples will be randomized to either TCU© or SUPPORT©, with equal allocation. Block randomization stratifying by gender of the adult with heart failure will be created by MPI CSL using an online research randomizer (www. randomizer.org). We will stratify by gender to maximize equal distribution between the two conditions. Both programs span 2 months in length and are delivered by trained interventionists to the couple via Zoom. All participants will be assessed at baseline (T1), post-treatment (T2), and 3 months post-treatment (T3) using separate self-report web-based measures. The study design is guided by the Consolidated Standards of Reporting Trials (CONSORT) extension criteria for pilot and feasibility trials [64] and the Standard Protocol Items: Recommendations for Interventional Trials (SPIRIT) guidelines—a SPIRIT checklist was completed [65]. The SPIRIT flow diagram of the trial is presented in Fig. 2. Patient recruitment and data collection began in July 2021 and is currently ongoing.

### Participants

Adults with heart failure are eligible to participate if they self-report a diagnosis of heart failure as an adult; currently experience heart failure symptoms or have been told they are NYHA Class II or III or AHA/ACC Stage C; are at least 18 years of age; have a spouse or partner they have lived with for at least 6 months that is willing and eligible to participate in the study; and have access to a device with a camera (e.g., computer, tablet) to participate in Zoom sessions, or a phone to participate in phone sessions. Partners (the term partner is used throughout the study to refer to both spouses and intimate partners) are eligible to participate in the study if they are also at least 18 years of age and have lived with the adult with heart failure for at least 6 months.

Couples are excluded if either member has major uncorrected hearing impairment, has significant cognitive impairment, is enrolled in another intervention trial that would prevent them from completing the requirements of this study, and has active psychosis or severe substance abuse that would impair their ability to complete the study. Couples will also be excluded if the adult with heart failure has had a heart transplantation, is in receipt of a mechanical circulatory support device, or has another terminal illness that would prevent them from participating in a five-month study. Couples are not required to be married and no couples will be excluded based on sexuality or gender identity.

### Recruitment and data collection procedures

Couples are recruited through the Tufts Medical Center Heart Failure clinic, Boston, Massachusetts, and also through community outreach locally (e.g., local newspapers, councils on aging, newsletters) and nationally via clinical partners in cardiology clinics, websites (e.g., Family Caregiver Alliance) and social media (e.g., Facebook). Study investigators at Tufts Medical Center will communicate with eligible patients in their heart failure clinic about the study and provide them with the study recruitment materials that include the study phone number at Boston College and QR code managed by the study research team at Boston College. Interested participants contact the Boston College study team either by phone or the QR code and are screened for eligibility by phone. Similarly, our community outreach recruitment involves distributing information through media, social media, and presentations, and interested participants are provided information to call, email, or use the QR code to contact the Boston College study team for more information and/or to schedule a time for a phone eligibility screening. Eligible and interested couples are e-mailed separate links to provide consent using a web-based survey, though participants are provided the option to complete paper versions of the consent form by mail. Couples are considered to be formally enrolled and consented when electronic or written consent forms are completed by both members of the couple.

Participants complete three surveys over the course of the study at baseline, month 2 (post-treatment), and month 5 (3 months post-treatment). Adults with heart failure and their partners complete separate surveys. Participants can complete the surveys either by Research Electronic Data Capture (REDCap)—a HIPAA-compliant, secure, web-based application—or by mail survey. This allows participants to have greater choice for their preferred mode, which increases both recruitment and retention across the adult lifespan. In rare circumstances, we will also provide a phone interview to complete the survey. All participating couples will be assigned a code number and all identifying information will be removed from all data sources (electronic and hard copy). All study data will be stored in a secure, password-protected network folder on the Boston College server. Data from mail surveys will be entered and verified by trained research staff before merging with data obtained via REDCap. Due to minimal risk of the study, MPIs are responsible for data monitoring.

Upon completing the baseline survey, couples are randomized by the project coordinator to either the TCU© arm or the SUPPORT© arm and receive a phone call from their assigned interventionist to inform them of their assigned program and to schedule the first session. Upon completion of the study, couples receive a $100 gift card. In cases where one or both members of the couple cannot or does not want to continue participating in their assigned program (TCU© or SUPPORT©), either member of the couple may still choose to complete follow-up data assessments, at approximately 2 and 5 months post-baseline. However, participants cannot continue in their assigned program as individuals (they must complete the program together).

### Intervention group

*Taking Care of Us©* is a theoretically and empirically informed intervention that is communication-based and relationship-focused, building on the strengths of the couple while fostering new skills. Drawing upon our theory of dyadic illness management [31], previous research, and key components of successful dyadic and self-management interventions, the goals of the seven session program are to improve shared symptom appraisal and dyadic management by fostering communication, collaboration, and confidence within the couple, thereby leading to better dyadic health. The sessions are designed to encourage participants to reflect on their strengths and areas of challenge as a couple and to rephrase their goals from individual to dyadic. Throughout the sessions, the interventionist works with the couple and facilitates communication and problem-solving to reach shared goals and strategies that balance the health and needs of both members. Tools are used to summarize and enhance discussions and agreed-upon strategies and goals. Each of the seven sessions last approximately 45–60 min and take place by zoom or phone. Sessions occur approximately once a week starting approximately 1 week after the baseline surveys are complete (the entire program is approximately 2 months in length).

Content builds throughout the program and is tailored to the area couples agree to work on together, reflecting a respect for each couple’s readiness to change. Early on the importance of respectful and supportive communication is introduced with specific skills the couple can practice. Strong emphasis is placed on speaker-listener techniques so that members of the couple can share and hear in a facilitated context. The program also places emphasis on the social aspect of confidence and role of supporting one another in increasing confidence as a team. Because the program is strengths-based, it provides couples with skills, confidence, and knowledge to collaborate and support one another, and communicate with each other to balance their respective health needs and take a team approach to living with heart failure. Couples will practice skills and try out simple strategies between sessions and then reflect on how things went with the interventionist and each other to brainstorm when changes would help. The goal of such exercises is to encourage the couple to practice skills learned in sessions and demonstrate the manageable way goals can be broken down into small and simple strategies. Successful strategies will be noted and combined with a shared collaborative plan the couple creates during the sessions that will be sent to the couple on completion of the program. The seven sessions and their content and format are presented in Table 1.

**Table 1:** Taking Care of Us© Intervention session overview

Session	Session title	Session outline
1	A couple-based approach to heart failure	∙ Overview of the Taking Care of Us program ∙ Overview of the importance of heart failure symptom management ∙ Working as a team through communication
2	How are we doing?	∙ The positive roles of communication, collaboration and confidence ∙ Engaging in self-management activities
3	Living with heart failure part I	∙ Communicating about heart failure symptoms ∙ Recognizing heart failure symptoms
4	Living with heart failure part II	∙ Collaboration and support ∙ Shared goals for working together
5	Supporting care partner health	∙ Care partner health and needs ∙ Supporting the care partner
6	Strengthening our relationship	∙ Strengthening our relationship: fun activities, closeness, physical intimacy
7	Putting it all together	∙ Creating a long-term plan ∙ Staying flexible ∙ Involving healthcare providers and other family/support

Each couple will receive a *Taking Care of Us©* binder with materials to accompany each session (i.e., resource pages summarizing the theme of each session, worksheets to facilitate in-session activities related to each session’s theme, and home practice activities). The binder also includes local and national resources that may be helpful (e.g., heart failure, mental health, disability, financial, caregiving, housing, food).

### Control group

The SUPPORT© program is an attention-control condition and comprises of three sessions that occur approximately 1, 4, and 8 weeks after the baseline survey is completed (the program is also approximately 2 months in length to match the intervention exposure period). Each session is 45–60 min in length and takes place via Zoom or phone. The program is an educational counseling program similar to heart failure educational interventions currently available and is, therefore, a more realistic comparison condition than “usual care.” Although it is a class I guideline recommendation that all patients with heart failure should receive education and support to facilitate self-care [66], there are no agreed-upon standards and therefore significant variation among practices regarding the content, quality, and frequency of education delivered. As such, participants not randomized to the TCU© intervention receive basic education to harmonize minimally enhanced usual care as an active control. Additionally, all participants have access to non-study healthcare services during the study, and we would not have been able to ensure that participants would not be given certain elements of education with a traditional usual care group [67].

Session 1 overlaps with the first session of TCU© by focusing on heart failure symptoms and self-care of heart failure. Session 2 focuses on healthy eating, and session 3 focuses on physical activity. All sessions are designed to facilitate reflection and preliminary action planning and focus primarily on the person with heart failure (though the partner is required to be present in all sessions). Materials represent current clinical practice guidelines and publicly available educational information developed by the Heart Failure Society of America and the American College of Sport Medicine. Thus, the program is considered reproducible. Each couple will receive a SUPPORT© binder with materials to accompany each session (i.e., resource pages summarizing the theme of each session). The binder also includes the same local and national resources provided to the TCU© group (e.g., heart failure, mental health, disability, financial, caregiving, housing, food).

### Interventionists

Both programs are delivered by interventionists with a bachelor’s or master’s degree in mental health. Interventionists were trained by the study MPIs through a series of in-depth sessions guided primarily by the TCU© intervention manual and SUPPORT© manual, which both contain overviews of the respective program, guiding principles, scripts for each session, session materials (resource pages and activity sheets that were also provided to the participants), protocol for conducting the sessions and completing the session summary sheets and fidelity checklists, tips for dealing with challenging scenarios, and reporting adverse events or safety concerns. The TCU© and SUPPORT© manuals were both reviewed by two experts (one in heart failure and one in psycho-social dyadic interventions) that were not involved in the development of the manuals. Suggested revisions to the materials were made before the start of the pilot study.

Additionally, interventionists were provided with key background readings on heart failure, caregiving, and a dyadic approach to illness including the theoretical framework underpinning the intervention. Training sessions focused on the elements of the training manual mentioned above, the goals of each session, role-playing, dealing with challenging scenarios (i.e., disagreement and conflict), and confidentiality and safety. Over time, interventionists continue to be supervised with sessions reviewed and debriefed regularly. Interventionists are trained to deliver both programs to avoid unnecessary interventionist effects.

### Safety

The current pilot study has been deemed minimal risk and involves a communication-based, relationship-focused intervention compared to an educational program. All members of the team receive training on identifying and reporting adverse events (e.g., high emotional distress, high level of interpersonal conflict between members of the couple) or safety concerns (e.g., those mentioned in the consent form related to threats of physical harm to oneself or others) to the MPIs promptly. Each member of the research team also receives a research training manual outlining all elements of the research protocol and requesting that adverse events and safety concerns identified during interactions with participants (e.g., during phone follow-ups, delivery of sessions) be reported to the MPIs immediately for determination of action. Interventionists are also required to document adverse events and safety concerns brought up by participants during sessions on the session summary sheet and also reported to the MPIs immediately. Given the minimal risk of the study, a safety monitoring committee consisting of both MPIs and two leading researchers in the fields of heart failure and caregiving research was formed to oversee and monitor any safety concerns or adverse events that might arise during the study. All adverse events will be promptly reported to the Boston College IRB and funding agency.

### Fidelity

Treatment fidelity in the current study is maximized through the use of clear protocols and manuals that are scripted, ongoing training and supervision throughout the study, and fidelity checks. Fidelity checks include the use of fidelity checklists that are completed at the end of every session by the interventionists to document whether key components of the session were delivered. Session summary sheets are also completed at the end of each session, by the interventionists, to document the engagement of the couple, any challenges encountered in delivering the content, and their role as facilitator. These checklists and summary sheets provide a basis for ongoing training and supervision and are reviewed by MPIs within 24 h of each session. Finally, approximately 5% of sessions will be recorded at random throughout the study. Both MPIs view each recording independently within three days of the session and independently rate the session using the fidelity checklist. Each MPI also prepares a report on what went well during the session and any places where room for improvement or feedback is warranted. MPIs then meet together to discuss any inconsistencies in their observations before meeting with the interventionist to discuss the session. Couples will have the option of opting out of the possibility of being selected to have a session recorded for fidelity purposes. When a session is selected and the couple agrees to the recording, the session is viewed by the principal investigators within 3 days and then destroyed.

### Measures

The aims of the current study will examine the feasibility and acceptability of the program and explore change in the outcomes of dyadic and individual health of the couple, dyadic symptom appraisal, and dyadic management.

#### Acceptability and feasibility

There will be several indicators of feasibility (i.e., refusal rate, number of sessions attended, completion of program, and completion of follow-up assessment) and acceptability (i.e., satisfaction with the program, usefulness of the information provided, relevance of the topics to the couple, likelihood of recommending the program to other couples with heart failure). We will also assess benefits and drawbacks in both closed-ended (e.g., “I learned a lot during these sessions,” “The program took more time than I wanted to spend”) and open-ended questions. Items and measures were drawn from prior studies by members of this team (CJW) [68, 69]. Additionally, in a series of closed and open-ended questions, the participants will be asked about length of sessions, time between sessions, # of sessions, and preferred delivery modality and an open-ended question about other suggestions for improvement.

#### Health

The psychometrically sound 10-item PROMIS Global Health short form [70] will be used as a general measure of QOL for couples. The measure includes specific ratings of physical, mental, and overall QOL. Depressive symptoms will be measured using the reliable and valid Center for Epidemiological Studies-Depression (CES-D) scale [71]. The scale asks participants to respond to 20 items regarding how they felt over the past week (e.g., I felt depressed) on a 0 (rarely or none of the time) to 3 (most or all the time) scale. Items are summed to create a total score (range from 0 to 60), with higher scores indicating greater depressive symptomology. Anxiety will be measured using the psychometrically sound Brief Symptom Inventory (BSI) subscale [72]. Participants are asked about feelings during the past 7 days and provides five response options ranging from 0 (not at all) to 4 (extremely). The Stanford Patient Education Research Center (PERC) Healthcare Utilization is a reliable and valid assessment of healthcare use that will be administered at baseline and T3 to collect physician, mental health, emergency room visits, and hospitalizations for both members of the couple [73]. The Multidimensional Caregiver Strain Index [74] is a highly reliable measure of physical, social and interpersonal strain, time constraints, and demands for partners that will be administered at each wave. The Kansas City Cardiomyopathy Questionnaire, a psychometrically sound 12-item Likert scale, will be used to measure heart failure-specific quality-of-life for adults with heart failure. Scores range from 0 to 100 with higher scores reflecting better QOL [75–77].

#### Dyadic appraisal

The couple’s appraisal of heart failure symptoms (i.e., dyspnea, pain, fatigue) will be assessed at all waves using well-established, reliable, and valid measures. Dyspnea will be measured with the 6-item dyspnea subscale of the Heart Failure Somatic Perception Scale [78]. The scale asks about how much the adult with heart failure was bothered by dyspnea during the last week on a 0 (not at all) to 5 (extremely bothersome) scale [79]. Pain interference will be measured using the 6-item PROMIS pain interference scale [80]. Fatigue will be measured using the 8-item PROMIS fatigue scale [81–83]. Dyadic appraisal scores for each symptom will be calculated using multilevel modeling (see analysis plan below).

#### Dyadic management

Dyadic management is a broad concept that captures the verbal and non-verbal strategies that help dyads manage and cope with illness [31, 84]. In the current trial, we focus on three aspects of dyadic management—communication, collaboration, and confidence, to manage heart failure.

Communication within the dyad will be examined using the dyadic coping measure, which consists of two subscales (active engagement and protective buffering) [85, 86]. Active engagement assesses the extent to which the person views their partner’s active involvement and support [85, 86]. Participants respond to five items using a Likert scale from 1 (never) to 5 (very often). Protective buffering assesses the extent to which the person views their partner’s use of hiding concerns and denying worries [85, 86]. Participants respond to six items using a Likert scale from 1 (never) to 5 (very often). Both scales have demonstrated reliability and validity [86].

Collaboration will be examined through both individual and shared engagement in managing heart failure and engagement in dyadic coping behaviors. Couple engagement in heart failure management will be measured using the management scores of the Self-Care of HF Index v6.2 (SCHFI) [87]. The SCHFI has 6 items that capture management. Response options range from 1 (not likely) to 4 (very likely). Scores are standardized to 0–100 with higher scores indicating greater engagement. In addition, couples will be asked to rate how much they collaborate regarding management of heart failure using the revised Stanford Chronic Disease Self-Management measure [88]. Couples are asked to rate their level of collaboration around six aspects of the illness (e.g., fatigue, emotional distress) on a 0 (no collaboration) to 10 (a great deal of collaboration) scale. A summary score is created by calculating the mean collaboration level across the six items. Finally, the common dyadic coping subscale from the well-established and validated dyadic coping inventory [89] will measure how much couples engage in collaborative coping. Participants respond to items (e.g., joint problem-solving, communication) on a 1 (very rarely) to 5 (very often) scale.

Finally, confidence to manage heart failure will be assessed using the Stanford Chronic Disease Self-Management measure [88]. Couples are asked to rate their level of confidence to manage heart failure around six aspects of the illness (e.g., fatigue, emotional distress) on a 0 (no confidence) to 10 (a great deal of confidence) scale.

#### Socio-demographic and clinical characteristics

Surveys will also ask couples for key background and socio-demographic data, including, age, gender, length of relationship, educational status, employment status, race/ethnicity, time since diagnosis, stage of heart failure, HFpEF vs. HFrEF status, current heart failure medications, other chronic illnesses, and impact of the coronavirus.

### Analytic plan

We will examine the feasibility and acceptability of the TCU© intervention in several ways. First, adherence to the program will be examined in proportion of couples who completed 5 of the 7 TCU© sessions. Feasibility will be achieved if ≥ 30% of eligible couples enroll in the study, ≥ 70% of assigned couples complete the TCU© program, and ≥ 80% complete the follow-up assessment. Acceptability of the TCU© program will be achieved if ≥ 80% of adults with heart failure and their partners report being satisfied/very satisfied with “the program overall” and “the usefulness of the information provided in sessions” and agree/strongly agree “they would recommend the program to other couples” and “looked forward to the sessions”. Results that fall short of these benchmarks will prompt refinement of the program before proceeding to a main trial. Additionally, quantitative items regarding the strengths and limitations of the program (e.g., length of sessions, number of sessions, time between sessions, delivery modality, skills covered, and goals of the program) as well as two open-ended questions about the benefits and drawbacks of the program will be examined to determine needed refinements.

Multilevel modeling (MLM) will be used to explore changes in outcomes between the two programs. MLM controls for interdependencies of the dyadic data and autocorrelations among repeated assessments. We will run a series of longitudinal models to explore the role of an indicator variable (GROUP) on adult with heart failure and partner changes in dyadic health, dyadic management, and dyadic appraisal variables over time. A significant coefficient for GROUP on the slope parameter will indicate the rate of change across time is statistically dependent on treatment condition. For individual level health variables (i.e., care strain and heart failure specific-QOL) individual MLM will be used to examine the role of GROUP. We will also have the option, particularly if we under-recruit, to conduct analysis of covariance to explore change in outcomes between both programs for adults with heart failure and partners separately. Due to the pilot nature of the study and primary focus on feasibility and acceptability, we will conduct two-sided statistical tests with *p* < 0.05 without correction for multiple tests.

### Sample size justification

Although there is consensus that pilot studies should have primary focus on feasibility and acceptability, there is less clarity regarding the sample size needed with ranges of 10–75 per group [90]. Recent work has focused on decision points to move forward to a main trial (green zone), need for refinement (amber zone), and unacceptable outcome (red zone). With a sample size of 60 couples, we will be able to estimate a completion rate (or other benchmark stated above) of 80% to within a 95% confidence interval of ± 10%. If the sample size drops to 40 couples, we can estimate an 80% completion rate to within a 95% confidence interval of ± 12%. Given our stated feasibility and acceptability benchmarks for moving forward, our proposed sample of 60 couples (30 per group) is deemed adequate to evaluate feasibility and acceptability and results of any statistical tests will not be used for hypothesis testing [90].

## Discussion

This program is the first known heart failure dyadic intervention that will target specific, modifiable aspects of the dyadic relationship (i.e., collaboration, communication, and confidence) to foster skills to enhance the way couples appraise and manage heart failure together. The program is tailored to the strengths and challenges for each couple and facilitates the creation of shared goals for each couple and the strategies to obtain them as a team. Our program is dyad-based (i.e., includes both members of the couple) and dyad-focused (i.e., targets the outcomes of both members and facilitates a collaborative approach to management needs of each member) [31]. We will evaluate the feasibility and acceptability of the program and explore changes in outcomes. This pilot study is the first step to advance understanding of heart failure management within the context of a couple and begin to address the lack of interventions that optimize the outcomes of both the adult with heart failure and their partner [10].

## Data Availability

The datasets generated and/or analyzed during the current study are not publicly available due to confidentiality but may be available from the corresponding author on reasonable request.

## Abbreviation

TCU: Taking Care of Us

## References

[1] Dharmarajan K, Wang Y, Lin Z, Normand ST, Ross JS, Horwitz LI (2017). Association of changing hospital readmission rates with mortality rates after hospital discharge. JAMA.

[2] Benjamin EJ, Virani SS, Callaway CW, Chamberlain AM, Chang AR, Cheng S (2018). Heart disease and stroke statistics-2018 update: a report from the American Heart Association. Circulation.

[3] Denfeld QE, Winters-Stone K, Mudd JO, Gelow JM, Kurdi S, Lee CS (2017). The prevalence of frailty in heart failure: a systematic review and meta-analysis. Int J Cardiol.

[4] Lee CS, Mudd JO, Hiatt SO, Gelow JM, Chien C, Riegel B (2015). Trajectories of heart failure self-care management and changes in quality of life. Eur J Cardiovasc Nurs.

[5] Buck HG, Harkness K, Wion R, Carroll SL, Cosman T, Kaasalainen S (2015). Caregivers' contributions to heart failure self-care: a systematic review. Eur J Cardiovasc Nurs.

[6] Timonet-Andreu E, Morales-Asencio JM, Alcala Gutierrez P, Cruzado Alvarez C, Lopez-Moyano G, Mora Banderas A (2020). Health-related quality of life and use of hospital services by patients with heart failure and their family caregivers: a multicenter case-control study. J Nurs Scholarsh.

[7] Luttik ML, Blaauwbroek A, Dijker A, Jaarsma T (2007). Living with heart failure: partner perspectives. J Cardiovasc Nurs.

[8] Evangelista LS, Stromberg A, Dionne-Odom JN (2016). An integrated review of interventions to improve psychological outcomes in caregivers of patients with heart failure. Curr Opin Support Palliat Care.

[9] Srisuk N, Cameron J, Ski CF, Thompson DR (2016). Randomized controlled trial of family-based education for patients with heart failure and their carers. J Adv Nurs.

[10] Buck HG, Stromberg A, Chung ML, Donovan KA, Harkness K, Howard AM, Kato N, Polo R, Evangelista LS (2018). A systematic review of heart failure dyadic self-care interventions focusing on intervention components, contexts, and outcomes. Int J Nurs Stud.

[11] Bradley EH, Curry L, Horwitz LI, Sipsma H, Thompson JW, Elma M (2012). Contemporary evidence about hospital strategies for reducing 30-day readmissions: a national study. J Am Coll Cardiol.

[12] Kitko L, McIlvennan CK, Bidwell JT, Dionne-Odom JN, Dunlay SM, Lewis LM (2020). Family caregiving for individuals with heart failure: a scientific statement from the American Heart Association. Circulation.

[13] Riegel B, Dunbar SB, Fitzsimons D, Freedland KE, Lee CS, Middleton S (2021). Self-care research: where are we now? Where are we going?. Int J Nurs Stud.

[14] Yancy CW, Jessup M, Bozkurt B, Butler J, Casey DE, Drazner MH (2013). 2013 ACCF/AHA guideline for the management of heart failure: a report of the American College of Cardiology Foundation/American Heart Association Task Force on Practice Guidelines. Circulation.

[15] Lee CS, Gelow JM, Denfeld QE, Mudd JO, Burgess D, Green JK (2014). Physical and psychological symptom profiling and event-free survival in adults with moderate to advanced heart failure. J Cardiovasc Nurs.

[16] Lee CS, Hiatt SO, Denfeld QE, Mudd JO, Chien C, Gelow JM (2015). Symptom-hemodynamic mismatch and heart failure event risk. J Cardiovasc Nurs.

[17] Freedland KE, Hesseler MJ, Carney RM, Steinmeyer BC, Skala JA, Davila-Roman VG (2016). Major depression and long-term survival of patients with heart failure. Psychosom Med.

[18] Rutledge T, Reis VA, Linke SE, Greenberg BH, Mills PJ (2006). Depression in heart failure: a meta-analytic review of prevalence, intervention effects, and associations with clinical outcomes. J Am College Cardiol.

[19] Lyons KS, Hiatt S, Gelow JM, Auld J, Mudd JO, Chien CV (2018). Depressive symptoms in couples living with heart failure: the role of congruent engagement in heart failure management. Aging Ment Health.

[20] Lyons KS, Hutton-Johnson S, Lee CS (2021). The role of symptom appraisal, concealment and social support in optimizing dyadic mental health in heart failure. Aging Ment Health.

[21] Jani BD, Mair FS, Roger VL, Weston SA, Jiang R, Chamberlain AM (2016). Comorbid depression and heart failure: a community cohort study. PLoS ONE.

[22] Vellone E, Chung ML, Cocchieri A, Rocco G, Alvaro R, Riegel B (2014). Effects of self-care on quality of life in adults with heart failure and their spousal caregivers: testing dyadic dynamics using the actor-partner interdependence model. J Fam Nurs.

[23] Allen JK, Dennison CR (2010). Randomized trials of nursing interventions for secondary prevention in patients with coronary artery disease and heart failure: systematic review. J Cardiovasc Nurs.

[24] Burke RE, Johnson-Loenke R, Nowels C, Silveira MJ, Jones J, Bekelman DB (2016). Can we engage caregiver spouses of patients with heart failure with a low-intensity, symptoms-guided intervention?. Heart Lung.

[25] Hooker SA, Grigsby ME, Riegel B, Bekelman DB (2015). The impact of relationship quality on health-related outcomes in heart failure patients and informal family caregivers: an integrative review. J Cardiovasc Nurs.

[26] Martire LM, Lustig AP, Schulz R, Miller G, Helgeson VS (2004). Is it beneficial to involve a family member? A meta-analysis of psychosocial interventions for chronic illness. Health Psychol.

[27] Hartmann M, Bazner E, Wild B, Eisler I, Herzog W (2010). Effects of interventions involving the family in the treatment of adult patients with chronic physical diseases: a meta-analysis. Psychother Psychosom.

[28] Sebern MD, Sulemanjee N, Sebern MJ, Garnier-Villarreal M, Whitlatch CJ (2018). Does an intervention designed to improve self-management, social support and awareness of palliative-care address needs of persons with heart failure, family caregivers and clinicians?. J Clin Nurs.

[29] Reid J, Ski CF, Thompson DR (2013). Psychological interventions for patients with coronary heart disease and their partners: a systematic review. PLoS ONE.

[30] Berg CA, Upchurch R (2007). A Developmental-Contextual model of couples coping with chronic illness across the adult life span. Psychol Bull.

[31] Lyons KS, Lee CS (2018). The theory of dyadic illness management. J Fam Nurs.

[32] Pucciarelli G, Vellone E, Savini S, Simeone S, Ausili D, Alvaro R (2017). Roles of changing physical function and caregiver burden on quality of life in stroke: a longitudinal dyadic analysis. Stroke.

[33] Streck BP, Wardell DW, LoBiondo-Wood G, Beauchamp JES (2020). Interdependence of physical and psychological morbidity among patients with cancer and family caregivers: review of the literature. Psychooncology.

[34] Wilson SJ, Pengb J, Andridgeb R, Jaremkac LM, Fagundesd CP, Malarkeye WB (2020). For better and worse? The roles of closeness, marital behavior, and age in spouses’ cardiometabolic similarity. Psychoneuroendocrinology.

[35] Badr H, Acitelli LK (2017). Re-thinking dyadic coping in the context of chronic illness. Curr Opin Psychol.

[36] Rohrbaugh MJ, Cranford JA, Shoham V, Nicklas JM, Sonnega JS (2002). Couples coping with congestive heart failure: role and gender differences in psychological distress. J Fam Psychol.

[37] Riegel B, Moser DK, Anker SD, Appel LJ, Dunbar SB, Grady KL (2009). State of the science: promoting self-care in persons with heart failure: a scientific statement from the American Heart Association. Circulation.

[38] Janssen JA, Spruit MA, Wouters EFM, Schols JMGA (2012). Symptom distress in advanced chronic organ failure: disagreement among patients and family caregivers. J Palliat Med.

[39] Quinn C, Dunbar SB, Clark PC, Strickland OL (2010). Challenges and strategies of dyad research: cardiovascular examples. Appl Nurs Res.

[40] Retrum JH, Nowels CT, Bekelman DB (2013). Patient and caregiver congruence: the importance of dyads in heart failure care. J Cardiovasc Nurs.

[41] Lee CS, Mudd JO, Auld J, Gelow JM, Hiatt SO, Chien CV (2017). Patterns, relevance and predictors of heart failure dyadic symptom appraisal. Eur J Cardiovasc Nurs.

[42] Lyons KS, Lee CS, Bennett JA, Nail LM, Fromme EK, Hiatt S (2014). Symptom incongruence trajectories in lung cancer dyads. J Pain Symptom Manage.

[43] Merz EL, Malcarne VL, Ko CM, Sadler M, Kwack L, Varni JW (2011). Dyadic concordance among prostate cancer patients and their partners and health-related quality of life: does it matter?. Psychol Health.

[44] Lyons KS, Lee CS (2020). The association of dyadic symptom appraisal with physical and mental health over time in care dyads living with lung cancer. J Fam Nurs.

[45] Gorman JR, Smith E, Drizin JH, Lyons KS, Harvey SM (2020). Navigating sexual health in cancer survivorship: a dyadic perspective supportive care in cancer. Support Care Cancer.

[46] Hornbuckle LM, Rauer A, Winters-Stone KM, Springer C, Jones CS, Toth LP (2021). Better together? A pilot study of romantic partner influence on exercise adherence and cardiometabolic risk in African-American couples. J Racial Ethn Health Disparities.

[47] Lyons KS, Zajack A, Greer M, Chaimoy H, Dieckmann N, Carter JH (2021). Benefits of a self-management program for the couple living with Parkinson’s disease: a pilot study. J Appl Gerontol.

[48] Winters-Stone KM, Lyons KS, Dobek J, Dieckmann NF, Bennett JA, Nail L (2016). Benefits of partnered strength training for prostate cancer survivors and spouses: results from a randomized controlled trial of the exercising together project. J Cancer Surviv.

[49] Falconier MK, Kuhn R (2019). Dyadic coping in couples: a conceptual integration and a review of the empirical literature. Front Psychol.

[50] Lee CS, Vellone E, Lyons KS, Cocchieri A, Bidwell JT, D'Agostino F (2015). Patterns and predictors of patient and caregiver engagement in heart failure care: a multi-level dyadic study. Int J Nurs Stud.

[51] Lyons KS, Sadowski T, Lee CS (2020). The role of concealment and relationship quality on patient hospitalizations, care strain and depressive symptoms in heart failure dyads. Eur J Cardiovasc Nurs.

[52] Trivedi R, Slightam C, Fan VS, Rosland AM, Nelson K, Timko C (2016). A couples' based self-management program for heart failure: results of a feasibility study. Front Public Health.

[53] Bandura A (1989). Human agency in social cognitive theory. Am Psychol.

[54] Lorig KR, Ritter P, Laurent DD, Yank V (2019). Building better caregivers: a pragmatic 12-month trial of a community-based workshop for caregivers of cognitively impaired adults. J Appl Gerontol.

[55] Lorig KR, Ritter P, Stewart AL, Sobel DS, Brown BWJ, Bandura A (2001). Chronic disease self-management program: 2 year health status and health care utilization outcomes. Med Care.

[56] Rohrbaugh MJ, Shoham V, Coyne JC, Cranford JA, Sonnega JS, Nicklas JM (2004). Beyond the "self" in self-efficacy: spouse confidence predicts patient survival following heart failure. J Fam Psychol.

[57] Goodman H, Firouzi A, Banya W, Lau-Walker M, Cowie MR (2013). Illness perception, self-care behaviour and quality of life of heart failure patients: a longitudinal questionnaire survey. Int J Nurs Stud.

[58] Lee CS, Suwanno J, Riegel B (2009). The relationship between self-care and health status domains in Thai patients with heart failure. Eur J Cardiovasc Nurs.

[59] Arnold R, Ranchor AV, DeJongste MJL, Koeter GH, Ten Hacken NHT, Aalbers R (2005). The relationship between self-efficacy and self-reported physical functioning in chronic obstructive pulmonary disease and chronic heart failure. Behav Med.

[60] Buck HG, Lee CS, Moser DK, Albert N, Lennie TA, Bentley B (2012). Relationship between self-care and health-related quality of life in older adults with moderate to advanced heart failure. J Cardiovasc Nurs.

[61] Benazon NR, Foster MD, Coyne JC (2006). Expressed emotion, adaptation, and patient survival among couples coping with chronic heart failure. J Fam Psychol.

[62] Lyons KS, Gelow JM, Hiatt S, Mudd JO, Auld J, Chien CV (2018). The role of dyadic confidence on engagement in heart failure care behaviors. Gerontologist.

[63] Lyons KS, Vellone E, Lee CS, Cocchieri A, Bidwell JT, D'Agostino F (2015). A dyadic approach to managing heart failure with confidence. J Cardiovasc Nurs.

[64] Eldridge SM, Chan CL, Campbell MJ, Bond CM, Hopewell S, Thabane L (2016). CONSORT 2010 statement: extension to randomised pilot and feasibility trials. BMJ.

[65] Chan AW, Tetzlaff JM, Altman DG, Laupacis A, Gotzsche PC, Krleza-Jeric K (2013). SPIRIT 2013 statement: defining standard protocol items for clinical trials. Ann Intern Med.

[66] Heidenreich PA, Bozkurt B, Aguilar D, Allen LA, Byun JJ, Colvin MM (2022). 2022 AHA/ACC/HFSA guideline for the management of heart failure: a report of the American College of Cardiology/American Heart Association Joint Committee on Clinical Practice Guidelines. Circulation.

[67] Freedland KE, Mohr DC, Davidson KW, Schwartz JE (2011). Usual and unusual care: existing practice control groups in randomized controlled trials of behavioral interventions. Psychosom Med.

[68] Whitlatch CJ, Judge K, Zarit SH, Femia E (2006). Dyadic intervention for family caregivers and care receivers in early-stage dementia. Gerontologist.

[69] Zarit SH, Femia E, Watson J, Rice-Oeschger L, Kakos B (2004). Memory club: a group intervention for people with early-stage dementia and their care partners. Gerontologist.

[70] Hays RD, Bjorner JB, Revicki DA, Spritzer KL, Cella D (2009). Development of physical and mental health summary scores from the patient-reported outcomes measurement information system (PROMIS) global items. Qual Life Res.

[71] Radloff LS (1977). The CES-D Scale: a self-report depression scale for research in the general population. Appl Psychol Meas.

[72] Derogatis LR, Melisaratos N (1983). The Brief Symptom Inventory: an introductory report. Psychol Med.

[73] Ritter PL, Stewart AL, Kaymaz H, Sobel DS, Block DA, Lorig KR (2001). Self-reports of health care utilization compared to provider records. J Clin Epidemiol.

[74] Stull DE (1996). The Multidimensional Caregiver Index (MCSI): Its measurement and structure. J Clin Geropsychol.

[75] Green CP, Porter CB, Bresnahan DR, Spertus JA (2000). Development and evaluation of the Kansas City cardiomyopathy questionnaire: a new health status measure for heart failure. J Am Coll Cardiol.

[76] Eurich DT, Johnson JA, Reid KJ, Spertus JA (2006). Assessing responsiveness of generic and specific health related quality of life measures in heart failure. Health Qual Life Outcomes.

[77] Spertus J, Peterson E, Conard MW, Heidenreich PA, Krumholz HM, Jones P (2005). Monitoring clinical changes in patients with heart failure: a comparison of methods. Am Heart J.

[78] Jurgens CY (2006). Somatic awareness, uncertainty, and delay in care-seeking in acute heart failure. Res Nurs Health.

[79] Jurgens CY, Fain JA, Riegel B (2006). Psychometric testing of the heart failure somatic awareness scale. J Cardiovasc Nurs.

[80] Amtmann D, Cook KF, Jensen MP, Chen WH, Choi S, Revicki D (2010). Development of a PROMIS item bank to measure pain interference. Pain.

[81] Christodoulou C, Junghaenel DU, DeWalt DA, Rothrock N, Stone AA (2008). Cognitive interviewing in the evaluation of fatigue items: results from the patient-reported outcomes measurement information system (PROMIS). Qual Life Res.

[82] Lai JS, Cella D, Choi S, Junghaenel DU, Christodoulou C, Gershon R (2011). How item banks and their application can influence measurement practice in rehabilitation medicine: a PROMIS fatigue item bank example. Arch Phys Med Rehabil.

[83] Stone AA, Broderick JE, Junghaenel DU, Schneider S, Schwartz JE. PROMIS fatigue, pain intensity, pain interference, pain behavior, physical function, depression, anxiety, and anger scales demonstrate ecological validity. J Clin Epidemiol. 2016;74:194-206. 10.1016/j.jclinepi.2015.08.029 26628334

[84] Lyons KS, Flatley C, Gorman JR, Hanan DM, Hayes-Lattin B (2022). Challenges experienced and resources identified by young to midlife couples 1–3 years post-cancer diagnosis. Psychooncology.

[85] Hagedoorn M, Kuijer RG, Wobbes T, Sanderman R (2000). Marital satisfaction in patients with cancer: does support from intimate partners benefit those who need it the most?. Health Psychol.

[86] Buunk BP, Berkhuysen MA, Sanderman R, Nieuwland W, Ranchor AV (1996). Actieve betrokkenheid, beschermend bufferen en overbescherming: meetinstrumenten voor de role van de partner bij hartrevalidatie [The role of the partner in heart disease: active engagement, protective buffering, and overprotection]. Gedrag Gezondheid.

[87] Riegel B, Lee CS, Dickson VV, Carlson B (2009). An update on the self-care of heart failure index. J Cardiovasc Nurs.

[88] Lorig KR, Stewart A, Ritter P, González V, Laurent D, Lynch J (1996). Outcome measures for health education and other health care interventions.

[89] Bodenmann G (2008). Dyadisches coping inventar: testmanual. [dyadic coping inventory].

[90] Lewis M, Bromley K, Sutton CJ, McCray G, Myers HL, Lancaster GA (2021). Determining sample size for progression criteria for pragmatic pilot RCTs: the hypothesis test strikes back!. Pilot Feasib Stud.

